# Concentrates of two subsets of extracellular vesicles from cow’s milk modulate symptoms and inflammation in experimental colitis

**DOI:** 10.1038/s41598-019-51092-1

**Published:** 2019-10-10

**Authors:** Abderrahim Benmoussa, Idrissa Diallo, Mabrouka Salem, Sara Michel, Caroline Gilbert, Jean Sévigny, Patrick Provost

**Affiliations:** 1 CHUQ Research Center/CHUL Pavilion, 2705 Blvd Laurier, Quebec, QC G1V 4G2 Canada; 2 Department of Microbiology, Infectious Diseases and Immunology, Faculty of Medicine, Université Laval, Quebec, QC G1V 0A6 Canada

**Keywords:** Digestive signs and symptoms, Ulcerative colitis, Experimental models of disease

## Abstract

Extracellular vesicles (EVs) are involved in cell-to-cell communication and modulation of numerous physiological and pathological processes. EVs are found in large quantities in milk and contain several inflammation- and immunity-modulating proteins and microRNAs, through which they exert beneficial effects in several inflammatory disease models. Here, we investigated the effects of two EV subsets, concentrated from commercial cow’s milk, on a murine model of colitis induced with dextran sodium sulfate (DSS). P35K EVs, isolated by ultracentrifugation at 35,000 g, and P100K EVs, isolated at 100,000 g, were previously characterized and administered by gavage to healthy and DSS-treated mice. P35K EVs and, to a lesser extent, P100K EVs improved several outcomes associated to DSS-induced colitis, modulated the gut microbiota, restored intestinal impermeability and replenished mucin secretion. Also, P35K EVs modulated innate immunity, while P100K EVs decreased inflammation through the downregulation of colitis-associated microRNAs, especially miR-125b, associated with a higher expression of the NFκB inhibitor TNFAIP3 (A20). These results suggest that different milk EV subsets may improve colitis outcomes through different, and possibly complementary, mechanisms. Further unveiling of these mechanisms might offer new opportunities for improving the life of patients with colitis and be of importance for milk processing, infant milk formulation and general public health.

## Introduction

Crohn’s disease (CD) and ulcerative colitis (UC) are the main forms of inflammatory bowel disease (IBD). They affect the gastrointestinal (GI) tract, which becomes severely damaged and inflamed, due to an abnormal response of the body’s immune system^1,2^ and deregulated homeostasis between the host and the microbiota^3^. The symptoms associated with these diseases are unpredictable and seriously burden daily life; abdominal pain/cramps, bloody diarrhea, incontinence, fatigue, recurrent hospitalizations and, in most urgent cases, surgeries^2,4,5^. The treatments currently available aim to reduce inflammation, which relieves symptoms and, in the best cases, leads to long-term remission^5^. However, the long-term use of these medications comes with important side effects, such as infections and kidney or liver damage^1^.

Extracellular vesicles (EVs) are small lipid membrane vesicles^6^ that carry bioactive proteins, cytokines and RNA, are involved in cell-to-cell communications^6,7^ and are associated with cancer and inflammation^8,9^. They are found in all biological fluids, with milk being the most enriched in EVs^10^. Milk is a complex fluid containing various EV subsets^11,12^, the most studied of which are exosomes. These small vesicles of ~100 nm in diameter are released when multivesicular bodies (MVB) fuse with the cell membrane^13^ and pellet at centrifugation speeds above 100,000 g (P100K). Recently, we discovered another EV subset in cow’s milk that sediments at a lower speed (35,000 g; P35K). Comparable to exosomes in terms of size and shape, but likely generated from budding of the cell membrane^11,14^, cow’s milk P35K EVs have a specific protein content and contain most microRNAs present in milk^12^.

Milk EVs are bioactive upon internalization or ingestion^15–18^. In 2012, Izumi *et al*.^19^ reported the resistance of raw milk microRNAs and exosomes to harsh chemical or enzymatic treatments. More recently, we reported that commercial cow’s milk EVs and part (10 to 15%) of their immunity-associated microRNAs (up to 10 billion copies of a single microRNA per 300 mL of milk) resisted simulated human digestion and were bioaccessible^20^. These results were confirmed by independent groups^21–23^, which also reported the internalization of milk EVs by intestinal epithelial cells within which they promoted differentiation and proliferation^21,22,24^. Milk EVs were also potent at preventing inflammation in two mouse models of rheumatoid arthritis^25^ and exerted beneficial effects in cancer^15^ – although they may increase inflammation under certain circumstances^26^.

These evidences led us to posit that, in a mouse model of dextran sodium sulfate (DSS)-induced colitis, ingested cow’s milk EVs may resist digestion *in vivo*, relieve the symptoms, tame inflammation and restore the integrity of the digestive tract by promoting cellular regeneration. The recently described interaction between bacteria and milk EVs^27,28^ also suggests that milk EVs may help manage colitis by mitigating microbial dysbiosis, which we aimed to investigate as a secondary outcome.

## Results

To verify our hypotheses, we compared the effects of two subsets of EVs isolated from commercial cow’s milk (P35K vs P100K milk EVs), that we had previously reported and characterized in depth^11,12,20^, in an experimental murine model of DSS-induced acute colitis. The first set of experiments involved 6 days of recovery after colitis induction (plan available as Supplementary Fig. S1A). Colitic and healthy control mice were fed, twice a day, either with the control solution (CS) or a feeding solution containing milk EVs sedimenting at 35,000 g (P35K EVs) or 100,000 g (P100K EVs).

### Milk EVs from P35K and, to a certain extent, P100K, improve recovery of DSS-treated mice

**Figure 1:**
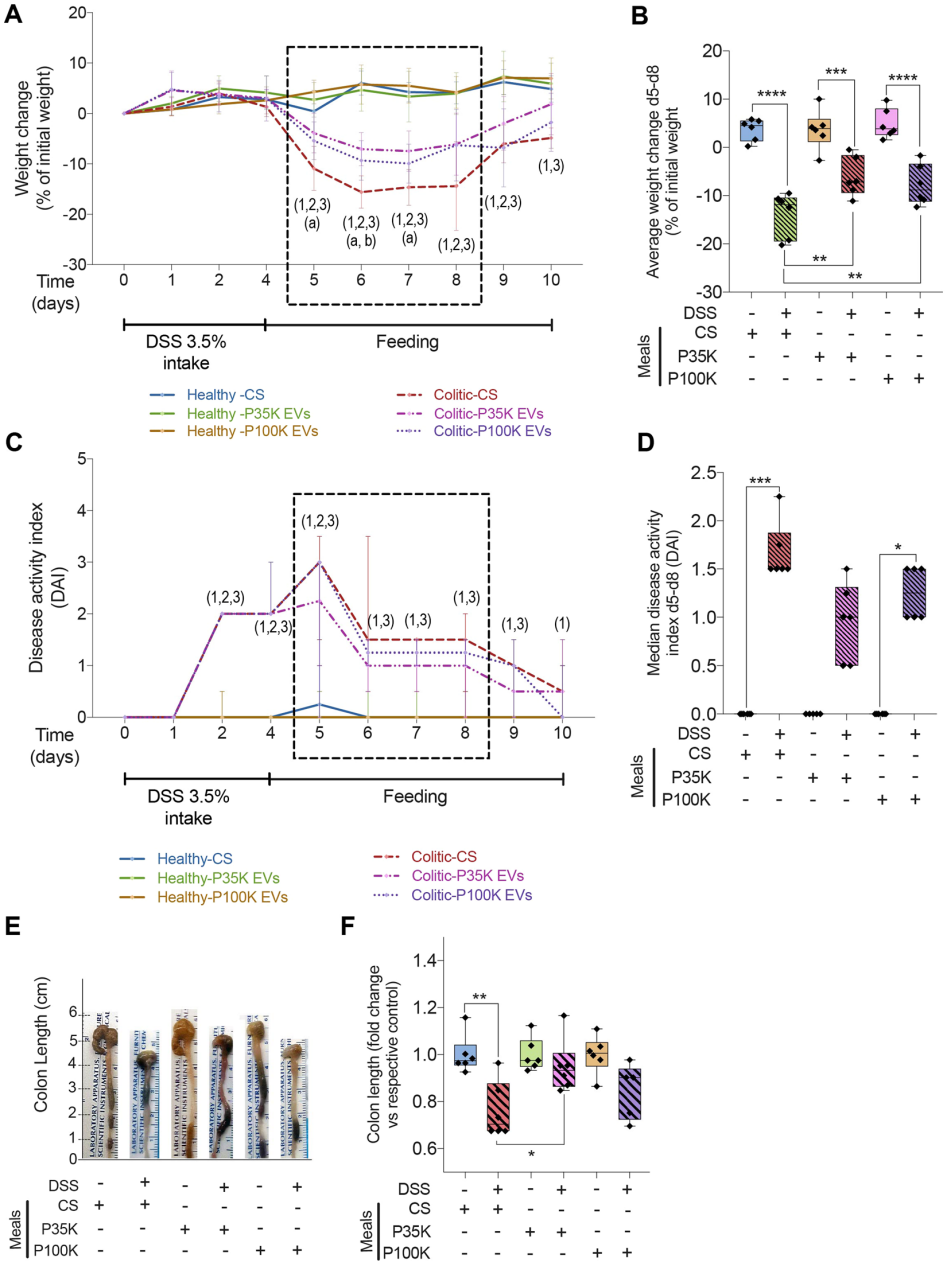
Milk EVs prevent weight loss and modulate disease activity during experimental colitis. Healthy and colitic mice were fed with one of the three feeding solutions (CS, control solution; P35K EVs; P100K EVs) for 6 days after disease induction. Healthy mice are in plain lines. Colitic mice are in dashed lines. (**A**) Weight change over time expressed as a percentage of the initial weight. (**B**) Average weight change at days 5–8 expressed as a percentage of initial weight. (**C**) Disease activity index (DAI) over time. (**D**) Median DAI between days 5 and 8. **Statistical comparison**. For weight, statistical significance was determined by one-way Anova with Bonferroni’s post-hoc correction for multiple comparison. For DAI, it was determined by Kruskal-Wallis one-way Anova with Dunn’s post-hoc test. For all tests, p < 0:05 was considered significant (n = 6/group). (**E**) Pictures of representative colons and caeca from healthy and colitic mice. (**F**) Colon length expressed as fold change versus respective control. **Significance display**. Numbers between brackets (1,2,3) indicate significant differences between healthy and colitic mice: (1) refers to mice fed with CS, (2) to mice fed with P35KEVs, and (3) to mice fed with P100K EVs. Letters (a,b) indicate significant differences between colitic mice fed with CS and colitic mice fed with P35K EVs (a) or with P100K EVs (b). *p < 0.05; **p < 0.01; ***p < 0.001, ****p < 0.0001. **Abbreviations**. CS, control solution; DAI, disease activity index; DSS, dextran sodium sulfate; d5-d8, day 5 today 8; EVs, extracellular vesicles; P100K, pellet 100,000 g; P35K, pellet 35,000 g.

Healthy mice showed a comparable weight gain during the experiment, independently of the solution they were fed with, suggesting that milk EVs do not influence weight under normal physiological conditions. DSS-treated mice receiving the CS significantly lost 10 to 15% of their initial weight, with their lowest weight levels observed between day 5 and day 8 (Fig. 1A,B).

EVs from P35K and, to a certain extent, P100K significantly tempered weight loss during the first two days of recovery (between day 4 and day 6) (Fig. 1A). The greatest differences were observed between day 5 and day 8, during which colitic mice fed with milk EVs (P35K or P100K) had significantly higher relative weight than those fed with the CS. Yet, they did not fully recover their initial weight (Fig. 1B). After day 8, all colitic mice started to recover their initial weight; only the mice fed with P35K EVs were comparable in weight to healthy control mice at day 10 (Fig. 1A,B). These results suggest that milk EVs, especially those from P35K, prevented weight loss associated with colitis and accelerated weight recovery.

Disease activity index (DAI) levels of healthy control mice were null during the entire experiment, independently of the feeding solution (Fig. 1C). No adverse effect linked to CS or milk EV treatments were observed.

All colitic mice reached a DAI level of 2 after 4 days of DSS intake (Fig. 1C). Between day 5 and day 6, EVs from P35K and, to a certain extent, those from P100K reached a DAI lower than colitic mice fed with the CS (Fig. 1C). Between day 5 and day 8, P35K EVs-treated mice had a DAI level no more different from its respective control (Fig. 1D). After 6 days of recovery (day 10), DSS-treated mice fed with either milk P35K or P100K EVs had reached a DAI comparable to healthy mice more quickly than those fed with CS (Fig. 1C).

There was a marked ~20% diminution in colon length in the colitic mice fed with the CS compared to healthy controls (Fig. 1E,F), which translates colitis-induced colon damage and cell loss. Feeding colitic mice with P35K EVs significantly prevented this diminution in colon length (Fig. 1E,F). Notably, when mice were sacrificed, we noted that mice fed with P35K EVs (either healthy or colitic) had colon qualified as “stiffer” and “denser” compared to the other mice. A similar tendency was observed with P100K EVs with no significative difference in colon length between these groups and heathy controls (Fig. 1E,F). However, P100K EV-fed mice were also comparable to the colitic group fed with CS, which advocates for partial recovery (Fig. 1E,F).

These results support the beneficial effects of milk P35K EVs and, to a certain extent, P100K EVs in restoring weight and diminishing the signs and symptoms of DSS-induced colitis.

### Milk EVs alleviate colon dysbiosis in colitic mice

In preliminary experiments, DSS-induced colitis caused dysbiosis (Supplementary Fig. S3A), as expected from the literature^29^. In another set of preliminary experiments, healthy mice fed with milk EVs had higher levels of certain bacterial strains than those fed with CS (Supplementary Fig. S3B). Interestingly, the phyla that were down regulated by colitis were the ones up regulated by milk EVs (Supplementary Fig. S3A,B).

**Figure 2:**
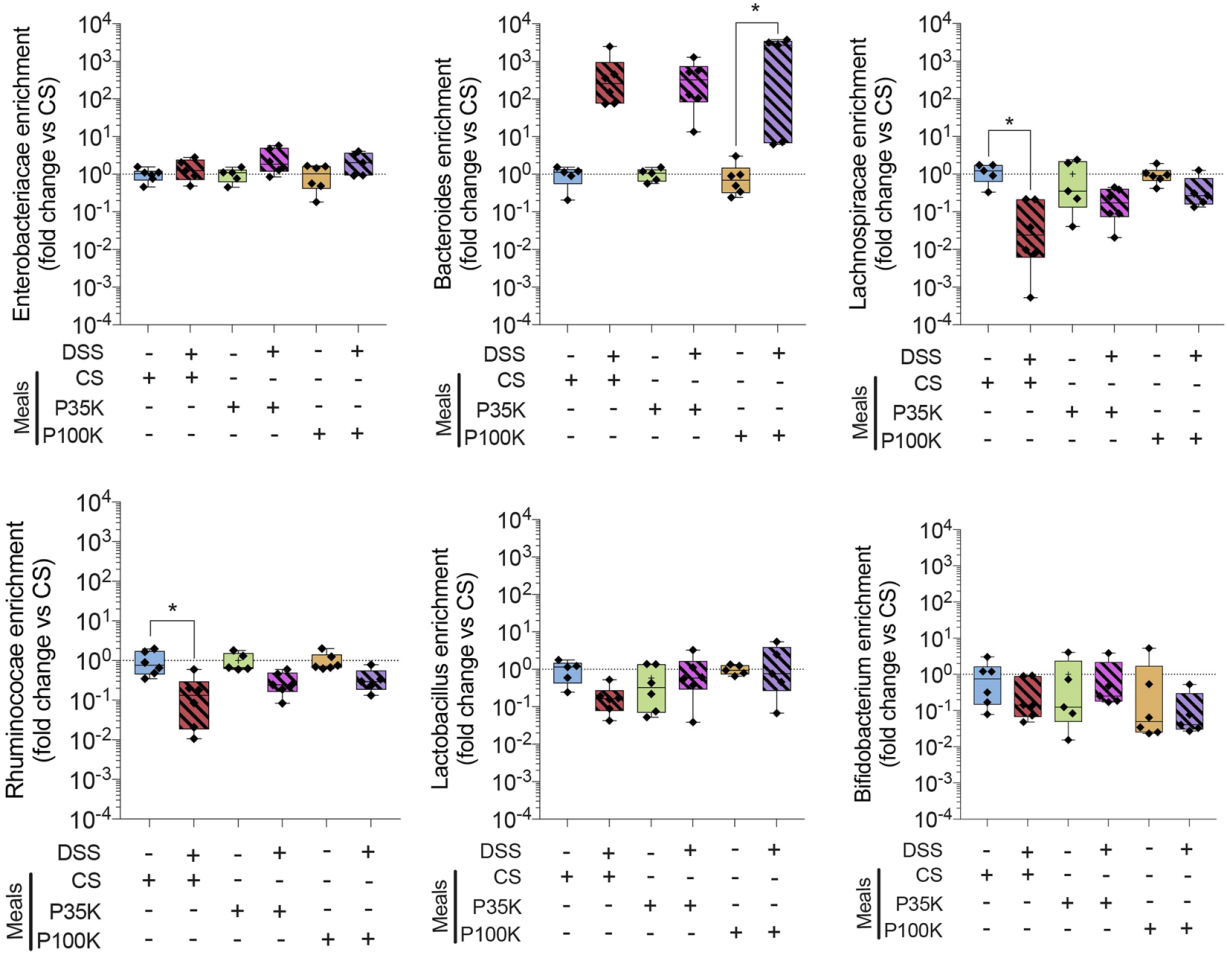
Milk EVs help manage colitis-induced colonic dysbiosis. Relative quantification of 6 colonic bacterial families by RT-qPCR. Data are expressed as fold change versus respective control ± SD. **Statistical comparison**. Statistical significance was determined by one-way Anova with Bonferroni’s post-hoc correction for multiple comparison. p < 0:05 was considered significant (n = 6/group). **Significance display**. *p < 0.05. **Abbreviations**. CS, control solution; DSS, dextran sodium sulfate; EVs, extracellular vesicles; FITC, fluorescein isothiocyanate; P100K, pellet 100 000 g; P35K, pellet 35,000 g; SD, standard deviation.

Therefore, we supposed that milk EVs could restore a normal gut bacteria profile after DSS-induced colitis. To verify this, we quantitated, by RT-qPCR, these bacterial strains in the faeces of colitic and healthy mice (Fig. 2D). There was a 2,000 to 4,000-fold increase in *Bacteroidetes-Prevotella* strains in all colitic groups, compared to respective healthy controls, but statistical significance was only reached for colitic mice fed with 100K EVs (Fig. 2D). There was no significant difference between healthy and colitic mice for *Enterobacteriacae*, *Lactobacillus* (including *Pediococcus/Leuconostoc spp*.) and *Bifidobacterium spp*. phyla, independently of the feeding solutions, suggesting that these strains are neither impacted by DSS-induced colitis nor by milk EVs (Fig. 2D). *Lachnospiracae* (*Clostridium* cluster XIVa) and *Ruminococaccae* (*Clostridium* cluster IV) were significantly downregulated by DSS-induced colitis (Fig. 2D), but their levels were restored, to levels comparable to healthy controls, by treating colitic mice with milk EVs (Fig. 2D).

These results are in accordance with our preliminary data (Supplementary Fig. S3B) and suggest that milk EVs may directly, or indirectly, restore normal levels of certain bacterial strains deregulated by colitis.

### Milk EVs restore normal colon histologic architecture and extracellular matrix secretion

**Figure 3:**
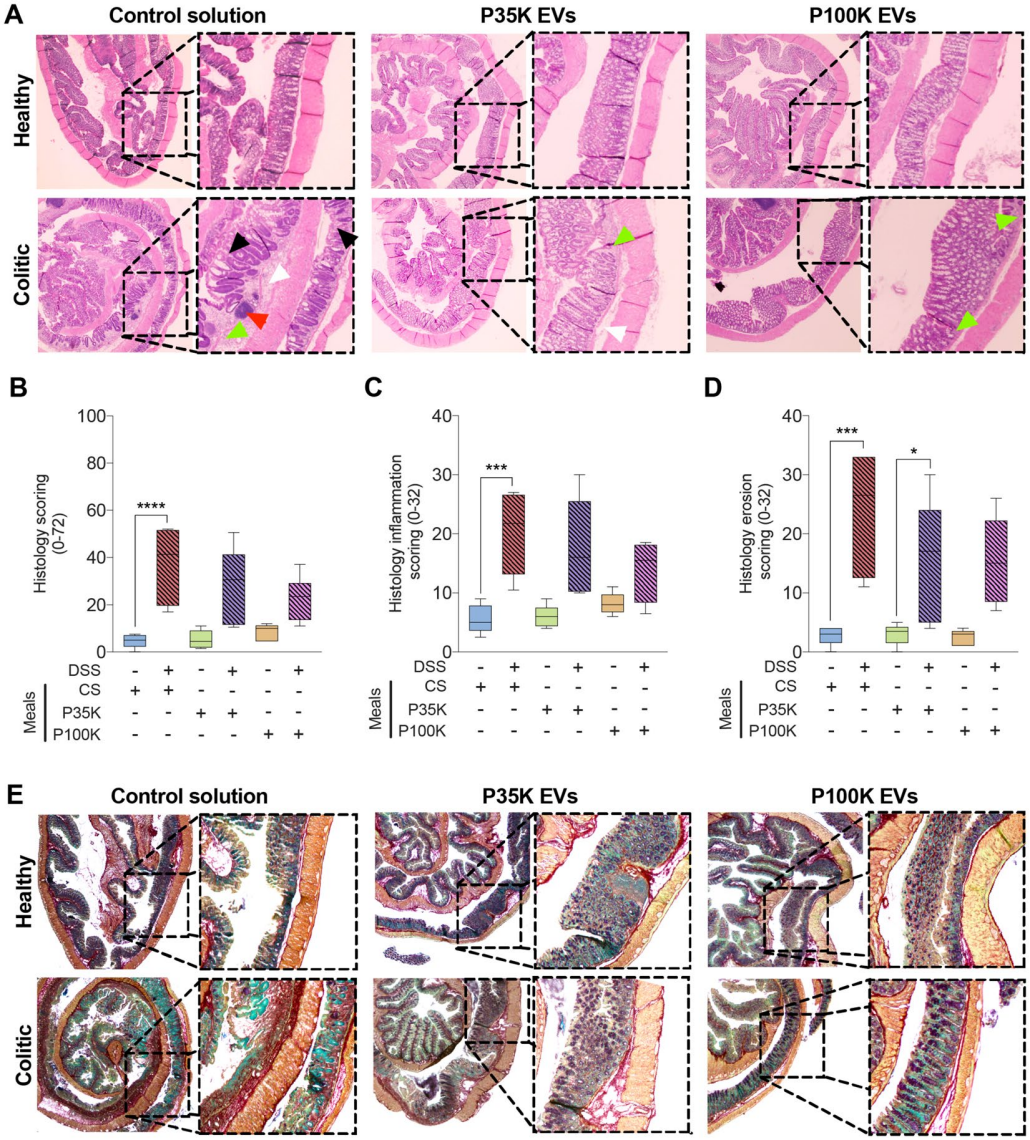
Milk EVs help restore colon histologic integrity, manage inflammation and regulate extracellular matrix (ECM) production. (**A**) Representative microscopy pictures of H&E-stained colonic sections from healthy and colitic mice fed with one of the three feeding solutions. White arrow heads indicate edema of the lamina propria, green point towards immune cell infiltration, black arrow heads indicate crypt erosion and red arrow indicates abscess. (**B**) Histopathological scoring of the disease (0–72). Data are expressed as median ± confidence interval (CI). (**C**) Histology scoring of inflammation (0–32). (**D**) Histology scoring of digestive wall erosion (0–32). (**E**) Representative microscopy pictures of pentachromic-stained colonic sections from healthy and colitic mice fed with one of the three feeding solutions. Muscles in orange, collagen in red, mucin in violet, IEC cells in green and red blood cells in yellow. **Statistical comparison**. Statistical significance was determined by Kruskal-Wallis one-way Anova with Dunn’s post-hoc test. P < 0:05 was considered significant (n = 6/group). **Significance display**. *p < 0.05; ***p < 0.001; ****p < 0.0001. **Abbreviations**. CI, confidence interval; CS, control solution; DSS, dextran sodium sulfate; ECM, extracellular matrix; EVs, extracellular vesicles; IEC: intestinal epithelial cells; H&E, hematoxylin and eosin; P100K, pellet 100,000 g; P35K, pellet 35 000 g; SD, standard deviation.

Colon sections were stained with Hematoxylin & Eosin (H&E) for histologic assessment and scoring (Fig. 3A–D) or with pentachromic stain^30^ to evaluate extracellular matrix (ECM) condition (Fig. 3E). Colitic mice treated with the CS had extensive colonic damage (i.e. ulceration of the mucosa, goblet cell depletion, crypt hyperplasia, edema) and immune cell infiltration (in the lamina propria and the mucosa), in comparison to healthy mice (Fig. 3A). This translated into a significantly higher histology score (Fig. 3B) that comprises a higher score of immune response (Fig. 3C) and of tissue erosion (Fig. 3D). This result is in accordance with the colon shortening observed after colitis (see Fig. 1).

There was no more significant difference between P35K EV-fed colitic mice and their respective healthy controls, for histology and immune cell infiltration scores, suggesting partial recovery of the tissue (Fig. 3A–D). However, erosion levels remained higher than healthy controls (Fig. 3D). P100K EVs were more potent than P35K EVs in preventing colon damage (Fig. 3A) and lowered all histology scores with no more significant difference between healthy and colitic mice (Fig. 3B-D).

Also, there was a loss of goblet cells’ mucin secretions (in violet surrounded by mucosa cells in green) in colitic mice fed with only the CS compared to healthy controls. There was also an increase in collagen secretion (in red), which suggests a starting fibrosis (Fig. 3E). Feeding with P35K EVs or P100K EVs restored mucins and collagen to levels comparable to healthy controls (Fig. 3E).

These results suggest that milk EVs might modulate inflammation and immune cell infiltration, prevent degradation of the colon and restore normal mucin secretion after colitis, with P100K EVs being slightly more potent at preventing inflammation and degradation of the tissue.

### Milk EVs restore gut barrier after two days of feeding

In the different experiments we performed, we monitored gut permeability in healthy and colitic mice at different time-points (day 0, 4, 7 and 10) by monitoring the translocation of dextran-FITC from the gut to the plasma. Because all healthy controls were always comparable, independently of the feeding solution, we choose to keep only one healthy control fed with the CS for this analysis.

**Figure 4:**
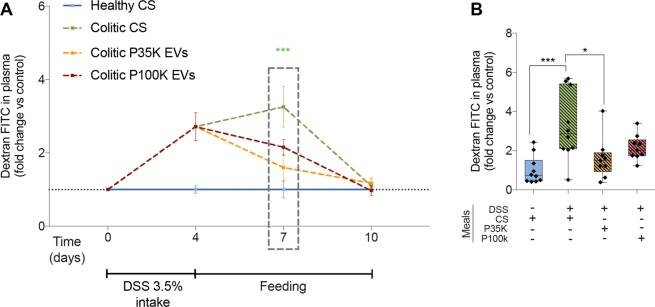
Milk EVs restore intestinal permeability *in vivo*. (**A**) Quantification of dextran-FITC in the plasma of healthy and colitic mice different times after feeding with one of the three feeding solutions. Fluorescence is expressed as fold change versus control. (**B**) Dextran-FITC in plasma at day 7. **Statistical comparison**. For all conditions, statistical significance was determined by one-way Anova with Bonferroni’s post-hoc correction for multiple comparison with p < 0:05 considered significant (n = 10/group). **Significance display**. *p < 0.05; **p < 0.01; ***p < 0.001. **Abbreviations**. CS, control solution; DSS, dextran sodium sulfate; EVs, extracellular vesicles; FITC, fluorescein isothiocyanate; P100K, pellet 100,000 g; P35K, pellet 35,000 g.

After the initial ~3-fold increase in gut permeability induced by colitis (days 0 to 4), all colitic mice returned to normal gut permeability level at the endpoint (Fig. 4A), with mice fed with milk EVs reaching it quicker (Fig. 4A). At day 7, gut permeability was significantly reduced in mice fed with P35K EVs, reaching levels comparable to healthy mice (Fig. 4A,B). P100K EVs also reduced gut permeability, though to a lesser extent, with no more significant difference with healthy mice. There was also no difference with the DSS-treated controls, suggesting a partial recovery of the digestive barrier upon ingesting P100K EVs (Fig. 4A,B). Therefore, two days of feeding with milk EVs restored the gut barrier lost after DSS-induced colitis.

### Milk EVs modulate inflammation in DSS-induced colitis

As the most important differences in weight, DAI and gut permeability were observed between day 5 and day 8, we analyzed the concentration of cytokines, chemokines and inflammation-related proteins in the colon of healthy and colitic mice sacrificed at day 7 (i.e. 2 days of feeding with milk EVs or the CS) using Bio-Plex technology.

**Figure 5:**
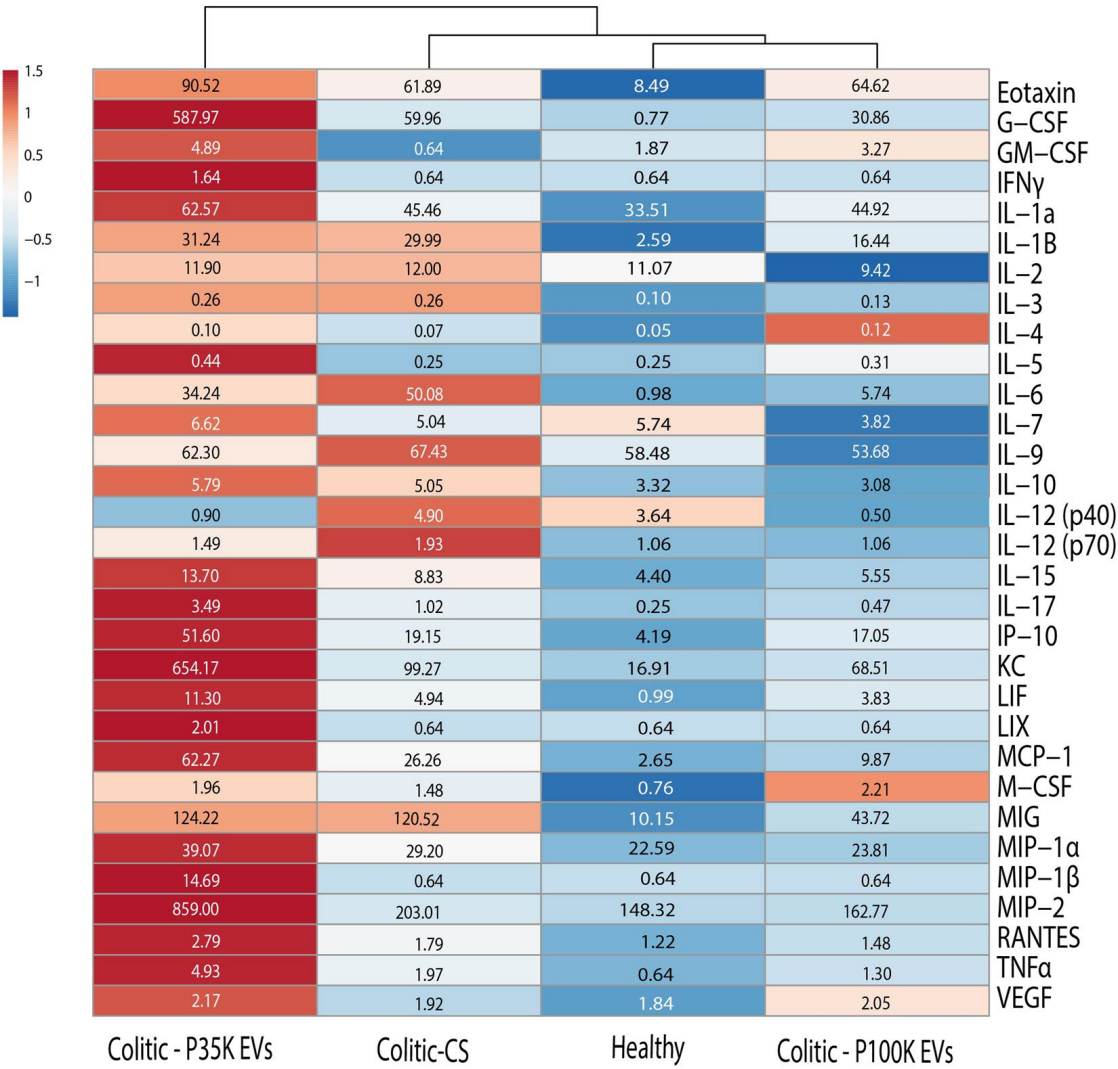
Milk EVs modulate cytokine production in the colon. Bio-Plex quantification of cytokines and chemokines in colon of healthy and colitic mice fed with milk EVs or the CS. Data are represented as a heatmap with clustering with median concentrations in pg/mL (inside the boxes) determined using a synthetic cytokine standard curve (n = 5/group randomly selected among n = 10 biological replicates). **Abbreviations**. CS, control solution; EVs, extracellular vesicles; P100K, pellet 100,000 g; P35K, pellet 35,000 g.

As expected, colitic control mice (Colitic-CS) had, compared to healthy controls, (i) higher levels of pro-inflammatory cytokines [IL-6 (+5,010%), IL-12 (p70, +82%), IL-1β (+1,057%), IL-15 (+100%), TNFα (+208%)], (ii) increased production of T-helper associated cytokines IL-3 (+60%) and IL-17 (+308%), and (iii) higher levels of the chemokines/innate immunity/immune cell proliferation-differentiation-survival modulators [CCL11 (Eotaxin, +629%), G-CSF (+7,687%), CXCL10 (IP-10, +357%), CXCL1 (KC, +487%), LIF (+399%), CCL2 (MCP-1, +891%), M-CSF (+95%) and CXCL9 (MIG, +1,085%)]. These results reflect a higher inflammation status and inflammatory response in colitic mice (Fig. 5, Healthy vs Colitic-CS). There was also a minor increase in IL-10 (+52%), IL-12 (p40,+34%) and in the chemokine CCL5 (Rantes, +46%). These results confirm that DSS-induced colitis increases colon inflammation, which persists after two days of recovery (Fig. 5, Healthy vs Colitic-CS).

Feeding colitic mice with P35K EVs led to a marked increase in chemokines and immune cell proliferation-differentiation-survival modulators Eotaxin (+46%), G-CSF (+880%), GM-CSF (+664%), CXCL10 (IP-10, +169%), CXCL1 (KC,+559%), LIF (+128%), CXCL5 (LIX,+214%), CCL2 (MCP-1, +137%), M-CSF (+32%), IL-7 (+31%), CCL3 and CCL4 (MIP-1α and MIP-1β, +33% and +2,195%, respectively), CXCL-2 (MIP-2, +323%) and CCL5 (Rantes, +55%) (Fig. 5, Colitic vs P35K-EVs). There was also a slight increase in proinflammatory cytokines IFN-γ (+156%), IL-17 (+242%) and TNF-α (+150%), and in IL-5 (+76%) (Fig. 5, Colitic vs P35K-EVs).

On the contrary, there was a marked decrease in pro-inflammatory IL-12-p40 (−444%), suggesting a lowering in inflammatory IL-23 levels^31–35^, a minor decrease in IL-12-p70 (−49%), with a slight increase in anti-inflammatory IL-4 (+30%) (Fig. 5, Colitic vs P35K-EVs).

With previous results, these data suggest that P35K EVs modulate colitis symptoms by promoting the development, and recruitment, of the innate immune system and encouraging immune cell proliferation-differentiation-survival towards a controlled cell-dependent inflammation.

When colitic mice were fed with P100K EVs, most of the cytokines/chemokines we monitored were lowered or restored to levels comparable to, or lower than, healthy controls (i.e. G-CSF, −48% versus Colitic-CS; IL-1β, −45%; IL-3, −50%; IL-6, −89%; IL-10, −39%; IL-12-p40, −90%; IL-12-p70, −45%; IL-15, −37%; IL-17, −53%; MCP-1, −62%; MIG, −63%; and TNFα, −34%). On the contrary, there was an increase in GM-CSF (+410%), anti-inflammatory IL-4 (+71%), IL-5 (+24%) and M-CSF (+49%) (Fig. 5, Colitic-P100K-EVs vs Colitic-CS).

Thus, P100K EVs might promote innate immunity and immune cell differentiation-proliferation-survival, but to a lesser extent than P35K EVs. Their major effect seems to be associated to cytokine production by diminishing the production/release of pro-inflammatory cytokines and increasing the production of anti-inflammatory ones. Clustering of the data further supports the restoration of normal cytokine/chemokine levels when colitic mice are fed with P100K EVs (Fig. 5).

Therefore, P35K EVs seem to mainly modulate the inflammation through regulation of the innate immunity and immune cell differentiation-proliferation-survival, while P100K EVs might tame inflammation by regulating the production/release of inflammatory/anti-inflammatory cytokines. Markedly, both EV subsets diminished IL-12-p40, suggesting interference in the production of the intestinal inflammation driver and pro-inflammatory cytokine IL-23^31–35^.

### P100K EVs down regulate colitis-associated microRNAs

**Figure 6:**
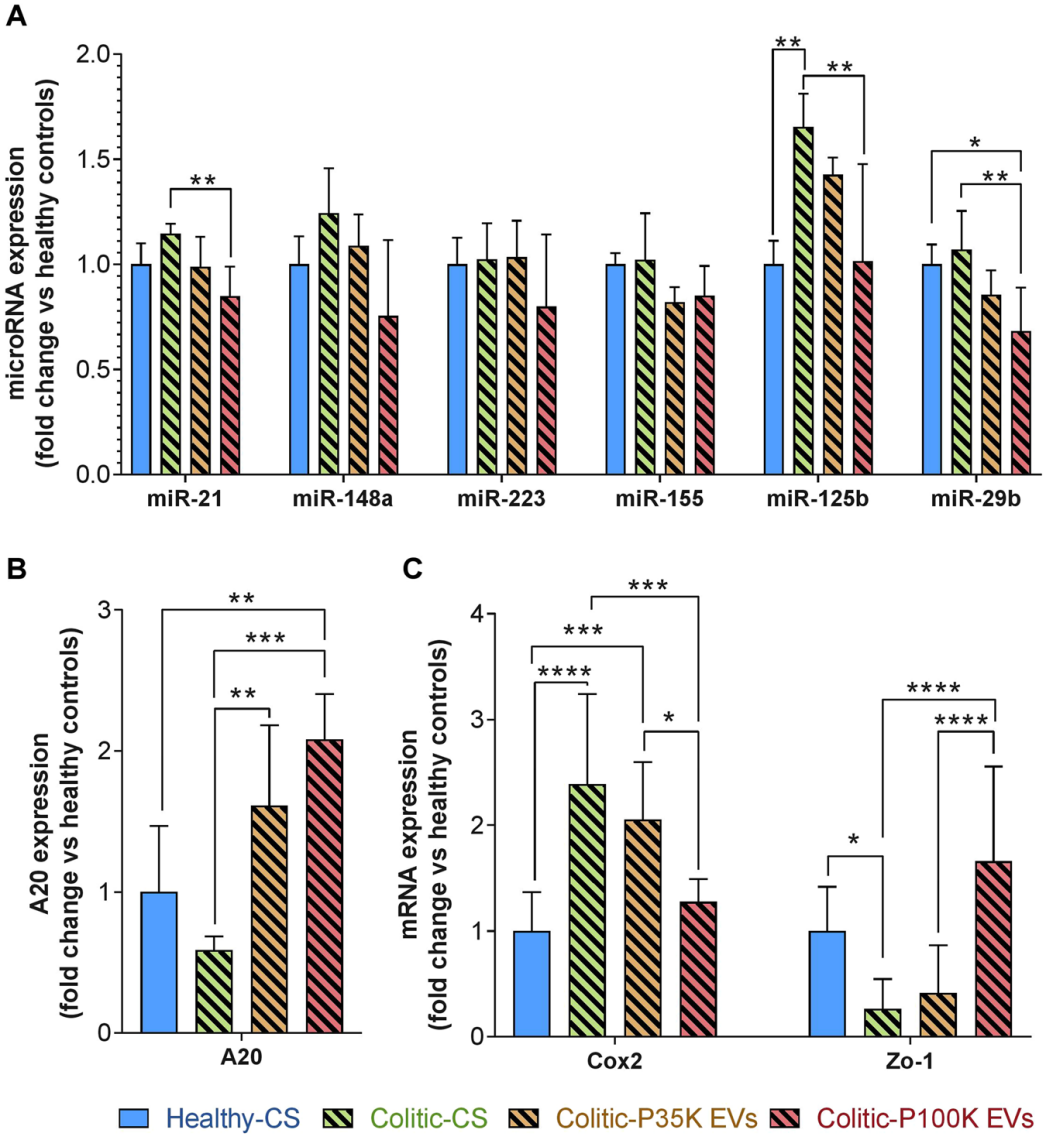
P100K EVs downregulated colitis-associated microRNAs, including miR-125b, enhanced the levels of the anti-inflammatory effector A20 and restored expression levels of COX2 and Zo-1. Quantification by RT-qPCR of colitis-associated microRNAs (**A**) or inflammation-associated genes (**B**,**C**) in colons of healthy and colitic mice. Data are normalized over RNU6 for microRNAs, RNA18S for mRNA, and reported over healthy controls using the ΔΔCq method. **Statistical comparison**. Statistical significance was determined by one-way Anova with Bonferroni’s post-hoc correction for multiple comparison with p < 0.05 considered significant (n = 10/group). **Significance display**. *p < 0.05; **p < 0.01, ***p < 0.001, ****p < 0.0001. **Abbreviations**. CS, control solution; EVs, extracellular vesicles; P100K, pellet 100,000 g; P35K, pellet 35,000 g.

We investigated the impact of milk EVs on colitis-related microRNAs in the colon (Fig. 6A). MiR-155 levels were stable in all conditions. P35K EVs did not impact significantly overall microRNA expression. MiR-21 decreased significantly in colitic mice fed with P100K EVs compared to colitic controls. The same profile was observed for miR-29b, whose expression was significantly lower in colitic mice fed with P100K EVs compared to healthy and colitic controls. Finally, and most interestingly, miR-125b expression increased in colitic mice fed with the CS, but was restored to normal levels after feeding colitic mice with P100K EVs (Fig. 6A). Overall, these results suggest that milk P100K EVs, but not P35K EVs, might resolve inflammation by restoring normal levels of immunity, inflammation and colitis-associated microRNAs miR-21, miR-29b and miR-125b.

### P100K EVs increased expression of anti-inflammatory A20 and normalized the levels of COX-2 and ZO-1

Notably, miR-125b is a well-reported colitis-associated microRNA that is known to inhibit translation of the anti-inflammatory protein TNFAIP3 (A20)^36^. This protein is central in regulating NFκB-induced inflammation, cell-junction and cytokine production/release^37–41^. There was a slight, yet not significant, decrease in the expression of A20 in colitic mice fed only with CS (EV-free solution). However, both EV subsets increased the levels of A20 (+135% with P35K EVs, +375% with P100K EVs), with P100K EVs being more potent than P35K EVs (Fig. 6B). P100K EVs also prevented the increase of COX-2 expression and restored the expression of ZO-1, which was diminished by DSS-induced colitis (Fig. 6C).

We found that P35K EVs, and P100K EVs, increased the expression of the anti-inflammatory protein A20, with P100K EVs specifically regulating NFκB-induced COX-2 expression. P100K EVs also restored levels of ZO-1 known to be downregulated in digestive inflammation through the activation of NFκB^42^.

## Discussion

In this study, we found that cow’s milk P35K EVs and, to a certain extent, P100K EVs prevented weight loss, colon shortening, colonic tissue damage and restored normal ECM in a murine model of ulcerative colitis. These two EV subsets also favored some beneficial bacterial strains and preserved colon barrier integrity. Moreover, P35K EVs modulated inflammatory cytokines and favored the production of differentiation-proliferation-survival-related chemokines/mediators, while P100K diminished the production and release of inflammatory cytokines. Finally, both EV subsets restored high levels of A20, an anti-inflammatory inhibitor of NFκB, but only P100K EVs downregulated the expression of miR-125b, an inhibitor of A20, and led to lower levels of COX-2 and higher levels of ZO-1. We did not, however, investigate their effect in combination or in prophylaxis, as described for other food-derived products^43^.

Milk EVs are known to be immunity modulators that promote development of the immune system^15^ and might regulate innate immunity^11^, but in a way that remains to be clarified. P100K EVs or a mix of P35K and P100K EVs exerted anti-inflammatory effects^44–46^, limited oxidative stress^45,47^ and were beneficial in inflammation-related diseases, like rheumatoid arthritis^25^. A recent report suggested that milk EVs might also have a pro-inflammatory effect depending on environmental conditions^26^. This effect was associated to an increase in macrophage polarization to M1 phenotype^26^.

Here, we observed that both EV populations modulated innate immunity and the production of immune cell maturation/activation mediators. As the regeneration process requires immigrating innate immune cells, for remodeling of the extracellular matrix, and for resolving inflammation^48–50^, further investigations would be needed to assess if the effects of P35K EVs are meant to support regeneration. Another possibility would be that these EVs might sustain deleterious chronic inflammation upon repeated consumption^2^. As P35K EVs are the most enriched commercial milk EV subsets^12^, their long-term effects could be a public health concern. Their presence in milk-derived products would explain, in part, the deleterious effect of dairy consumption in IBD^51^; it might thus be important to discard them from dairy products destined to patients suffering from digestive inflammation.

In the meantime, P35K EVs had a mitigated effect on inflammatory cytokines, increasing some while decreasing others, like IL-12-P40, a subunit of high importance in the formation of IL-23 and for lymphocyte Th-17 responses^31–35^. Since this response is primordial in mouse models of inflammation, and that P35K EVs markedly improved the symptoms in our model of colitis, it is more likely that P35K EVs are beneficial modulators that promote immunity towards a better intestinal health status. This is supported by the fact that these EVs enhanced the expression of the anti-inflammatory protein TNFAIP3 (A20), a negative regulator of NFκB pathway^36^. However, it is not clear if this was specific to P35K EVs’ activity, or merely a byproduct of the increased release of TNF-α^37^. Moreover, these EVs did not impact the expression of COX-2 and ZO-1, two genes involved in inflammation and cell junction, respectively, and whose expression is deregulated through the NFκB pathway^42,52^. Therefore, and as there is very little data available on P35K in the literature, the exact mechanisms underlying the beneficial effect of P35K EVs are still not clear and warrant further investigations.

We found more solid ground when looking at the mechanisms through which P100K EVs may exert their beneficial activity. P100K EVs decreased the levels of different microRNAs that are of high importance in the intestinal barrier architecture and function^53^, and which are overexpressed in IBD and in inflamed colon mucosa^54–57^. The most interesting microRNA is miR-125b, which was overexpressed in colitic mice and restored to normal levels by P100K milk EVs. Concomitantly, there was a marked increase in A20 upon P100K EV treatment. Interestingly, miR-125b may reinforce inflammation, through the NFκB pathway, by inhibiting the translation of the anti-inflammatory A20^36^. Also, although A20 mRNA is highly expressed in colitis, its protein levels are kept low demonstrating a failure in its translation^37^. At the same time, A20 inhibitor miR-125b is highly expressed in IBD^54^. Therefore, P100K EVs might specifically regulate NFκB pathway, and prevent inflammation, by reducing miR-125b levels and relieving the inhibition of A20.

In our model, IL-9 increased during colitis and its levels were back to normal after feeding with P100K EVs. There was also a marked decrease in miR-21 levels upon feeding with milk P100K EVs. It was previously reported that the overexpression of miR-21 causes a decrease in intestinal barrier functions during IBD through the inhibition of the Rho GTPase RhoB^57^ and an increase in fibrosis during IBD^58^. Also, a decrease in miR-21 levels diminishes the susceptibility to DSS-induced colitis through interaction with the microbiota^59^. Participating to the etiology of IBD, overexpression of miR-21 in IBD has been associated to IL-9 secretion^60^. Therefore, some of the beneficial effects of P100K milk EVs on colitis might come from the reduction of the IL-9/miR-21/CLDN8 pathway, which would impact RhoB, reduce gut permeability and modulate the microbiota^59–61^.

The implication of miR-29b during colitis remains ambiguous. MiR-29b diminishes dendritic cells’ apoptosis and cytokine production^62^. However, its deletion exacerbates UC, while its overexpression helps managing the disease^63^. Whereas miR-29b is known to prevent inflammation^64^, it increases collagen production and fibrosis in IBD^65^. In turn, miR-29b expression increases the levels of IL-6, which prevents fibrosis but increases inflammation^65^. Here, P100K EVs diminished slightly the levels of miR-29b and reduced IL-6 levels. Because of such ambiguity, the involvement of miR-29b and IL-6 in the mechanisms underlying the effects of P100K EVs remains to be clarified.

The mechanisms by which P100K milk EVs inhibit inflammation-related microRNAs, especially miR-125b, remain unclear. P100K EVs are known to diminish IL-2 expression^46^, a cytokine known to be increased in IBD^66^, and induces overexpression of miR-125b in immune cells^67^. Moreover, milk EVs promote the differentiation of immune T cells towards the T regulatory type, while limiting the interferon pathways^46^. Most importantly, comparable P100K EVs prevented the anti-CD3 activation of T lymphocytes *in vitro*, suggesting that these EVs may prevent the antigen presentation through T-cell receptors, which may lead to overall lower inflammation and explain the decrease in inflammatory cytokines^46^. Based on these observations, and our present data, it is thus possible that such mechanism might be driven through the NFκB-miR-125b-A20 pathway.

MiR-125b is important for monocyte activity^68^ and is highly expressed in lymphoid stem cells, where it stimulates the development of lymphocyte lineage^69^. MiR-125b is also involved in the survival of hematopoietic stem cells and functions in the maintenance of lymphoid balance^69^. It is also expressed in epithelial cells, where it plays an important role in epithelium homeostasis^70,71^. Therefore, defining the relation between miR-125b, P100K EVs, NFκB, A20 and inflammation remains challenging and might depend on the cell type^71^. Further studies using antagomir-125b or miR-125b knock-out models would be required to properly assess the links between these entities during colitis.

Notably, milk P100K EVs are enriched in TGF-β^72^ and modulate T-lymphocyte development and differentiation *in vitro*^46^. TGF-β is a cytokine that regulates the T-lymphocyte maturation and T-helper lymphocyte balance (Th-17/T-reg)^73^. Depending on the context, it might promote pro-inflammatory Th-17 T-lymphocytes or anti-inflammatory T-reg^73^. For instance, higher and sustained levels of TGF-β might be deleterious in IBD^74^, but its downregulation in the gut results in UC^75^. Interestingly, a milk formulation enriched in TGF-β is commercially available to help manage IBD^76^. Therefore, these EVs may modulate adaptative immunity towards higher or lower inflammation, depending on the context and through TGF-β signaling.

During dissection, we noticed that the colon of mice fed with P35K milk EVs were stiffer than the other mice. Some of P35K EVs’ beneficial effects on colitis may thus be associated with a better preservation of colon tissue integrity, an increase of ECM secretion and a rapid diminution in colon permeability. This is supported by the restoration of mucin levels in colitic mice after feeding with P35 EVs and previous proteomic analyses suggesting that these EVs were likely to promote vesicular secretion^11^. A recent report describing the role of a mix of milk EVs in increasing mucins release in the intestinal lumen further supports such hypothesis^77^.

Previous studies have shown that a mix of milk EVs enhanced cell proliferation, especially in intestinal epithelial cells (IECs)^78^. Both milk EV subsets might then promote proliferation of intestinal cells, which might explain support their beneficial effect on colitis outcomes. Although such an effect would, in theory, increase the risk of colon cancer, P100K EVs were suggested to be anti-cancerous^15^. It is therefore of importance to further study these two EV subsets, alone or in combination, in cancer models to assess their effect on cancer.

In this study, milk EVs limited colitis-induced dysbiosis, possibly by acting as prebiotics^27^ or as a decoy^11,79,80^, as they restored normal levels of butyrate-producing firmicutes (*Lachnospiracae*, *Clostridium* cluster XIVa and *Ruminococaccae*, *Clostridium* cluster IV)^81,82^. Here, it is important to note that the qPCR analysis of bacterial diversity that we performed was limited to phylum level and did not consider viruses and fungi as part of the microbiota. More thorough, detailed investigations are required to document the overall effects of milk EVs on microbial diversity (e.g. shotgun sequencing). While being of limited resolution, this approach opens however the way for future analysis of bacterial functions and metabolomic studies, focusing especially on bacteria-derived butyrate^83,84^. Butyrate is an important bacterial metabolite implicated in gut wall health^85^. It is anticarcinogenic^86^ and anti-inflammatory^87,88^, and its levels are diminished in patients with UC^89,90^. Therefore, some of the beneficial effect of milk EVs might be through restoring the levels of butyrate-producing bacterial strains^91^. Importantly, butyrate is known to restore the levels of the anti-inflammatory protein A20^92^, further supporting the interplay between milk EVs and the miR-125b-A20-NFκB inflammatory pathway.

**Figure 7:**
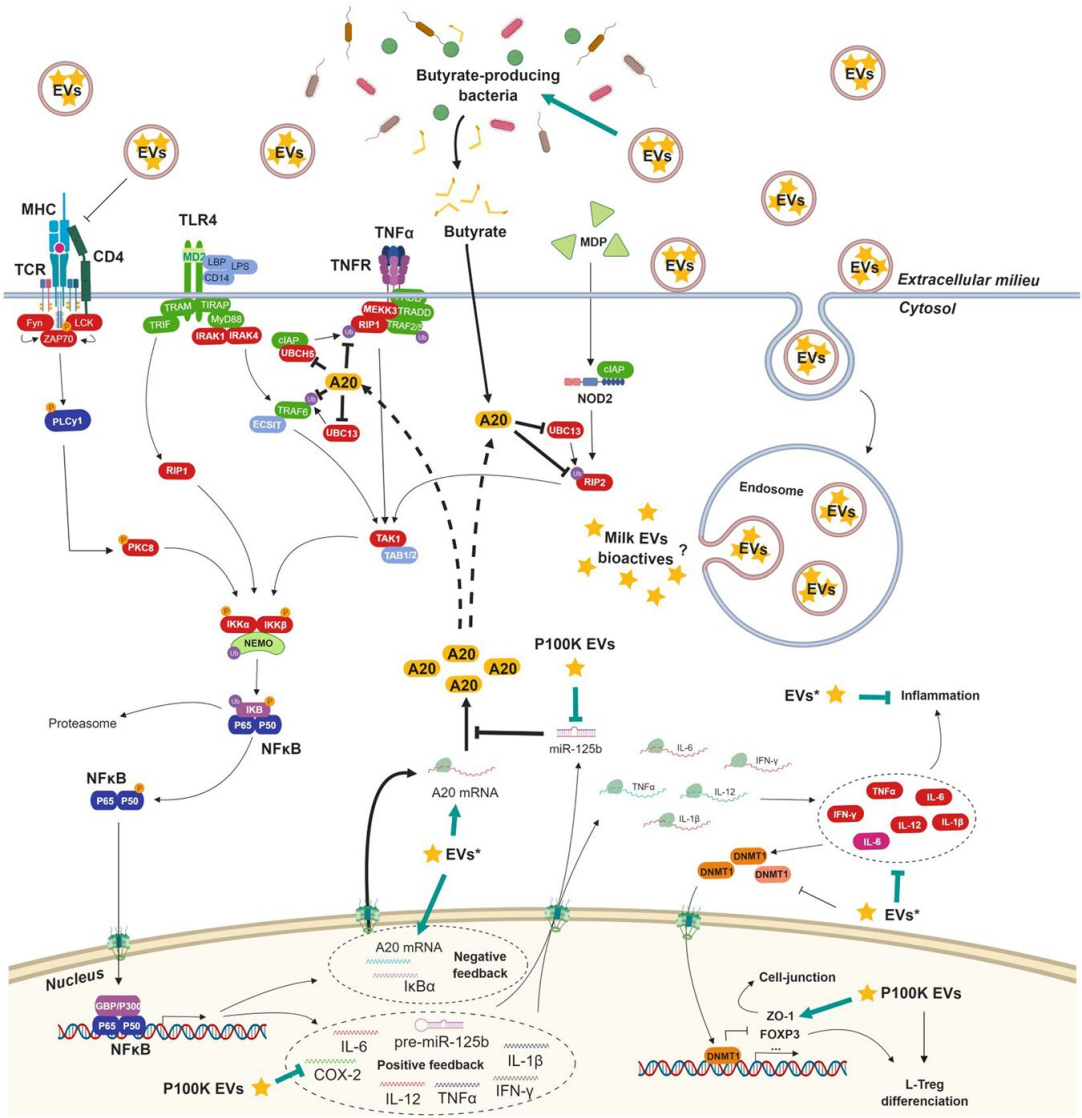
Putative and confirmed mechanisms through which milk EVs modulate inflammation. A20 plays a central role in regulating inflammation through the NFκB pathway. Milk EVs regulate A20 expression with P100K EVs specifically downregulating A20-inhibitor miR-125b. Black arrows represent previously reported interactions. Green arrows represent the new findings unveiled by the present study. More details are available in the discussion of this report. The bioactive compounds of milk EVs responsible for their bioactivity are yet to clarify, as is the mechanism ensuring their release in the cytoplasm. *Certain EV subset might be more potent than the others in the specified interaction. **Abbreviations**. EVs, extracellular vesicles; P100K, pellet 100,000 g; P35K, pellet 35,000 g. Made with BioRender.com.

As multiple outcomes of milk EV activities point towards a higher A20 expression, it is important that this protein is also involved in innate immune response through the inhibition of NOD2 signaling^40^. Moreover, it was also reported to promote cell-junction maintenance by relieving the inhibition of ZO-1 by NFκB^42^. The A20 protein may thus be central in milk EVs’ activity on IBD and other inflammatory diseases^38^, and possibly on cancer^39^. Future investigations should focus on the interplay between milk EVs and A20-related pathways. Figure 7 summarizes the discoveries we made on milk EVs and their anti-inflammatory properties through the regulation of miR-125b, A20, the microbiota and NFκB inflammatory pathway. It also includes previously reported links between milk EVs and inflammation, especially on the activation of T-cell receptor^46^ and DNA methyl-transferase 1 (DNMT1) inhibition^24^.

The caveat must be considered that the model we used here (DSS-induced colitis) only mimics the tissue damage associated with IBD with limited inflammation and no overreaction of the immune system as in naturally-occurring UC. Therefore, it would be important to replicate our findings in other animal models developing pathophysiological features closer to those observed in human patients (e.g. naïve T-lymphocyte transfer to Rag1−/− or MDR1a−/− transgenic mice)^93–95^.

Finally, although we have used supraphysiologic concentrations of milk EVs (10X), which, admittedly, limits extrapolation to milk consumption, it remains of utmost importance for the current development of milk EVs. Indeed, milk EVs have been suggested as potential oral vehicle for carrying and delivering different drugs to patients^96^. While their apparent biocompatibility^97^, survival to digestion^20^, resistance to harsh conditions^98^ and ability to load different compounds^99,100^, advocate for such a therapeutic use of EVs, it is important first to determine their intrinsic biological activity, especially on gut bacteria, inflammation and cancer, when milk EVs are formulated and administered as concentrates, rather than approaching this from a strictly nutritional perspective.

## Conclusion

The results reported in this study, along with the knowledge gathered so far^15^, suggest that milk EVs may modulate inflammation, colon bacteria dysbiosis, ECM secretion and cell proliferation, which, along with genetic disorders, contribute to the etiology of IBD. Different milk EVs had different, yet possibly synergetic, effects on the disease, especially through the interplay between NFκB and A20. Likely acting through the modulation of innate immunity, inflammatory response and microbiota, milk EVs might constitute a natural, biocompatible and widely available therapeutic asset in managing IBD and other inflammatory diseases. Our findings may also be of importance to children with malnutrition suffering from major gut damage or premature infants’ victim of necrotizing enterocolitis^77^. These new avenues of research warrant further investigations to assess the long-term safety of milk EVs and their impact on public health, and to define the ideal formulation for their possible use in clinic as a dietary approach, as drug carriers or as medial adjuvants. Replications, demonstration of the internalization of milk EVs and associated microRNAs, other mechanisms underlying milk EV function (e.g., on bacterial fermentation, receptor linking, etc.), and human trials are obviously necessary before drawing any conclusion on the beneficial or deleterious effects of milk EVs, and their content, on human health.

## Methods

### Dairy milk samples

We used commercial skimmed filtered dairy milk (Lactantia PurFiltre brand; http://www.lactantia.ca/food product/lactantia-purfiltre-skim-milk/) bought at a local grocery store in Quebec City, QC. Three milk tetra packs with different expiration dates were combined into one milk solution for isolating extracellular vesicles (EVs).

### Sedimentation of dairy milk extracellular vesicles (EVs) by differential ultracentrifugation

Milk EVs were obtained by following a previously described protocol, with slight modifications, that allows quick isolation of milk EVs with very little contaminating proteins [9, 15]. Two hundred (200) mL of dairy milk was mixed with 1 volume of 2% sodium citrate (in MilliQ water) that had been filtered with 0:22 µm membrane microfilters (Corning). The samples were subjected to successive differential ultracentrifugation steps at 35,000 g (35 K) for 2 h, then 70,000 g for 1 h, and 100,000 g (100 K) for 1 h at 4 °C in a Sorvall WX TL-100 ultracentrifuge, equipped with a SureSpin 630 Rotor (Sorvall). After each step, the pellets were suspended in 1 mL of 0:22 µm filtered sterile phosphate buffered saline (PBS) pH 7.4. Following our previous reports^11,12,20^, we chose to keep the EVs found in the 35,000 g pellet (P35K EVs) and the 100,000 g pellet (P100K EVs, often termed “exosomes”) and stored them at 4 °C overnight before preparing the feeding solutions. The MISEV2018 checklist^6^ was added as supplementary file.

### *In vivo* experiments

#### Ethical statement

This study was carried out in accordance with the guidelines, regulations and requirements of the Canadian Council of Animal Care for Animals Used for Scientific Purposes. All experiments were performed in accordance with the most up-to-date guidelines in a protocol approved by an independent ethical committee (Université Laval, Quebec, Canada). Well-being of the animals was monitored twice a day by trained animal facility services and any signs of distress or pain were immediately reported to the veterinary services. Mice losing more than 20% of their initial weight were sacrificed to prevent further suffering as per the ethical guidelines.

#### Mice

For all the experiments, we used 7 weeks-old male C57BL/6J mice (Charles Rivers, Montreal, Canada). Mice were randomly dispatched (3 to 5 mice per cage) with all groups (n = 6 or n = 10) containing mice comparable in weight (~22 g). Ulcerative colitis (UC) induction and treatments were performed after a 1-week acclimation period (mice were 8 weeks-old at the starting day of the protocol).

#### DSS colitis model

DSS colitis was induced following previously reported methodologies^43,101^. Acute colitis was induced by adding 3.5% (w/v) DSS (36–50 kDa, MP Biomedicals, USA) in sterile (autoclaved and filtered) drinking water for 4 days. The healthy controls were given the same sterile water used for preparing the DSS solution. Setup data are available as Supplementary Fig. S1.

#### Feeding solutions

Milk EV pellets were rinsed by dilution in 200 mL of the sterile vehicle solution (water:sodium citrate 2%; 1:1) and, after an overnight suspension, filtered with 0:22 µm membrane microfilters (Corning). EVs were then pelleted at their corresponding speed (35 K or 100 K) for 2 h at 4 °C in sterile conditions. Finally, the pellets were suspended in 50 mL of the vehicle solution and stored overnight at 4 °C before feeding. One dose (200 µL) of each feeding solution corresponded to EVs isolated from 10 mL of commercial cow’s milk (~430 mg/kg body weight).

The control solution (CS) was selected following the International Society of Extracellular Vesicles (ISEV) guidelines^6^. It corresponds to the supernatant (SN) of the last ultracentrifugation (100,000 g). It was chosen because it contains some contaminating proteins that could be confounders when studying milk EVs functionality. The SN was further depleted from EVs by ultracentrifugation (100,000 g, 16 h, 4 °C) and diluted with the sterile vehicle solution to match protein content of the milk EV feeding solutions. It is mostly composed of caseins and whey proteins^11^.

#### Mice feeding by gavage

Mice had ad libitum access to regular chow (Teklad global 19% protein, 2919) during the entire experiment. For the long-term experiments (10 days in total), there were 6 groups: 3 healthy controls, and 3 DSS-treated colitic groups. All were fed twice daily for 6 days with 200 µL of either the CS, P35K EVs or P100K EVs (Supplementary Fig. S2A). For the short-term experiment (6 days in total), there was a healthy control group and three DSS-treated colitic groups. The control groups were fed twice daily for 2 days with 200 µL of the CS or the milk EV preparations (Supplementary Fig. S2B).

#### Weight and disease activity index follow up

Weight (reported over the initial weight), stool consistency (normal solid stool, loose stool, diarrhea) and the presence of blood in the feces (no blood, gross bleeding) were consigned daily by the animal facility services blinded to the experiment template and allowed the determination of the disease activity index (DAI), following previously reported DAI scoring^43,101^.

#### Dextran-FITC gavage and measurements in plasma

Mice were starved overnight and fed by gavage with 100 µL of a fluorescein isothiocyanate (FITC)-labelled dextran solution (0.6 mg/g body weight, 4 kDa, Sigma, Oakville, Canada) diluted in sterile PBS filtered with 0:22 µm membrane microfilters (Corning) 4 h before sacrifice. Mice were anesthetized with Ketamine/Xylazine (10 mg/kg body weight) before blood puncture in the heart. Blood was stored in EDTA-coated tubes (50 µl EDTA 0.1 M/ml of blood) in the dark before plasma was isolated by two series of centrifugation at 3,500 g for 10 min at 4 °C. In parallel, a standard curve was prepared by diluting Dextran-FITC in sterile filtered PBS. The standard curve and the plasma samples were loaded on a black 96-well flat chimney plate (Corning) in duplicates before reading fluorescence (excitation, 488 nm; emission, 520 nm) in an Infinite 200 PRO plate reader (Tecan Life Science). Control mouse plasma with no FITC was used for fluorescence background.

#### Colon and colonic feces processing

After the sacrifice, the colon, along with the caecum, was carefully excised in sterile conditions (avoiding stretching the colon in the process). The colon’s length was immediately measured after excision (from caecum’s end to the distal region of the colon, considering the shape of the colon) by a technician blinded to the experimental design. The colon was then separated from the caecum, cut longitudinally and its fecal content was collected in sterile vials, flash frozen and later stored at −80 °C for microbiological analysis.

The colon was then carefully rinsed with sterile filtered PBS. The proximal sections of the colons (25 mg) were cut longitudinally and immediately flash frozen before storage at −80 °C for subsequent RNA isolation and RT-qPCR analysis. In the second round of experiments, the second sections were cut and stored at −80 °C for Bio-Plex cytokine quantification.

#### Colon preparation for histology staining

The remaining colon sections were carefully rolled (Swiss Roll), fixed in place with a needle and fixed in 4% PBS-buffered paraformaldehyde (PFA, Sigma, Oakville, Canada) overnight at 4 °C. Colons were then stored in 0.2% PBS-buffered PFA at 4 °C for less than a week. Finally, the tissues were rinsed and placed in a cassette for embedding in paraffin and processed for histologic staining (Hematoxylin & Eosin, H&E or pentachromic stain^30^) by the IBIS histopathology platform of the CHU de Quebec-Université Laval.

#### Histology scoring

Histologic disease scoring was performed by three trained scientists, blinded to the experiment template, on randomized samples and following previously reported methods^43,101^. Briefly, each slide was randomly assigned a code number (Random.org) and was then analyzed for eight factors translating digestive tract erosion and inflammation. Each of these factors was assigned a score ranging from healthy tissue (0) to severely damaged (3) and were multiplied by a factor expressing the extension of the damage from rare (1) to extensive (3). The addition of the factorized scores gave a total histologic scoring (0–72) subdivided into two scores for inflammation (0–32) and digestive wall erosion (0–32).

### Molecular biology analyses

#### RNA isolation

These steps were performed by two students blinded to the experiment design on randomized samples. Frozen colon sections (25 mg) were immediately soaked in RNA lysis buffer from the High Pure RNA-Tissue kit (Roche) without thawing the tissues. The tissues were lyzed using sterile 800-µm low-binding sterile silica beads (OPS Diagnostics) and a mini bead beater (GlenMills). Total RNA was isolated following manufacturer recommendations. After elution, 1 µg total RNA was used for reverse transcription using the MiScript RT^2^ kit (Qiagen).

#### RT-qPCR

After diluting the cDNA (1/10), RT-qPCR was performed using the SSo Advanced SYBR Green mix (Bio-Rad) in 96-well plates (MicroAmp™) using the StepOnePlus^TM^ device (Fisher Scientific) and specific primers (for microRNAs, MiScript Primer Assay, Qiagen; for mRNA, previously reported primers, IDT^43^). MicroRNA expression was normalized using the ∆∆Cq method^102^ and using, as a housekeeping genes, small nucleolar RNA RNU6, for microRNAs, or RNA18S for mRNAs^43,103^.

#### Cytokine quantitation of the colon sections

Frozen tissues were homogenized with sterile 800-µm low-binding sterile silica beads (OPS Diagnostics) and a mini bead beater (GlenMills) in cold sterile filtered lysis buffer (Tris 45 mM, pH 7.4, NaCl 95 mM, PMSF 0.1 M) supplemented with one complete Mini EDTA-free Protease Inhibitor Cocktail Tablet (Roche Diagnostics) per 10 ml. The volume of lysis buffer was defined using the following formula: 8 × weight tissue in mg = volume of lysis buffer in µl. Once tissues were homogenized, the whole was centrifuged at 10,000 g for 10 min at 4 °C to discard the debris and the beads. The supernatant was transferred to a new tube and samples were normalized by dilution in the lysis buffer to achieve the same protein concentration (1,214 mg/mL) before storage at −80 °C.

Colonic cytokine expression was determined using a multiplex immunoassay and standard curves for each cytokine (Bio-Plex 200 Mouse Cytokine Array/Chemokine Array 32-Plex, Millipore MILLIPLEX) at the Eve Technologies Corporation platform (Calgary, Canada).

#### Colonic bacteria quantitation by qPCR

Total DNA was extracted from mouse stool specimens using PowerSoil DNA Isolation Kit (MOBIO, Catalog No. 12888-50) following the manufacturer’s procedures with 800 µm low binding silica beads (OPS Diagnostics) and a bead beater to disrupt the feces. After total DNA isolation, specific primers were used to monitor bacterial species of the gut microbiota in healthy or colitic mice. RT-qPCR analysis was conducted using SsoAdvanced^TM^ Universal SYBR Green Supermix real-time PCR reagent (Bio-Rad, Catalog No. 1,725,270) and StepOnePlus^TM^ Real-Time PCR System (Bio-Rad, Catalog No. 4,376,600). Data were normalized over the total bacteria primer, which is designed to amplify all bacterial strains (Eubacteria), using the ∆∆Ct method^102,104^. All primers and annealing temperatures used for bacterial quantitation are indicated in Supplementary Table S1.

### Statistical analysis

All statistical analyses were performed using R (Free Software Fondation) and Prism 7 (Graph-Pad Software, Inc.). All experiments were conducted in biological replicates (n = 6 or 10) following power calculations for the selected tests using G*Power^105^ software (alpha set at 0:05 and 1-beta set at 80%) using standard deviation and effect size data from previously reported work^43,106–112^ and our pilot studies. For all experiments, the first type error alpha was set to 0:05 (5%) with p value below 0:05 considered significant. After normal distribution assessment (Shapiro-Wilk test), statistical significance was determined by one-way Anova with Bonferroni’s post-hoc correction for multiple comparisons or by Kruskal-Wallis one-way Anova with Dunn’s post-hoc test for non-parametric data.

### Figures and illustrations

Figures displayed in this manuscript were generated using R (Free Software Foundation) along with Inkscape software (Free Software Foundation) and Prism 7 (GraphPad Software, Inc.).

## Supplementary information

Supplementary Information File

## Data Availability

The datasets generated and/or analyzed during the current study have been displayed, are provided as supplementary files or are available from the corresponding author on a reasonable request.
