# Supplementary material for: Concentrates of two subsets of extracellular vesicles from cow’s milk modulate symptoms and inflammation in experimental colitis
Source: Sci Rep. 2019 Oct 10;9:14661. doi: 10.1038/s41598-019-51092-1 (PMC6787204; doi:10.1038/s41598-019-51092-1)
Supplement: Supplementary file 1 — Supplementary Information File [file 41598_2019_51092_MOESM1_ESM.pdf]

SUPPLEMENTARY FIGURES

Concentrates of two subsets of extracellular vesicles from cow's milk modulate symptoms and inflammation in experimental colitis

Abderrahim Benmoussa, Idrissa Diallo, Mabrouka Salem, Sara Michel, Caroline Gilbert, Jean Sévigny & Patrick Provost*

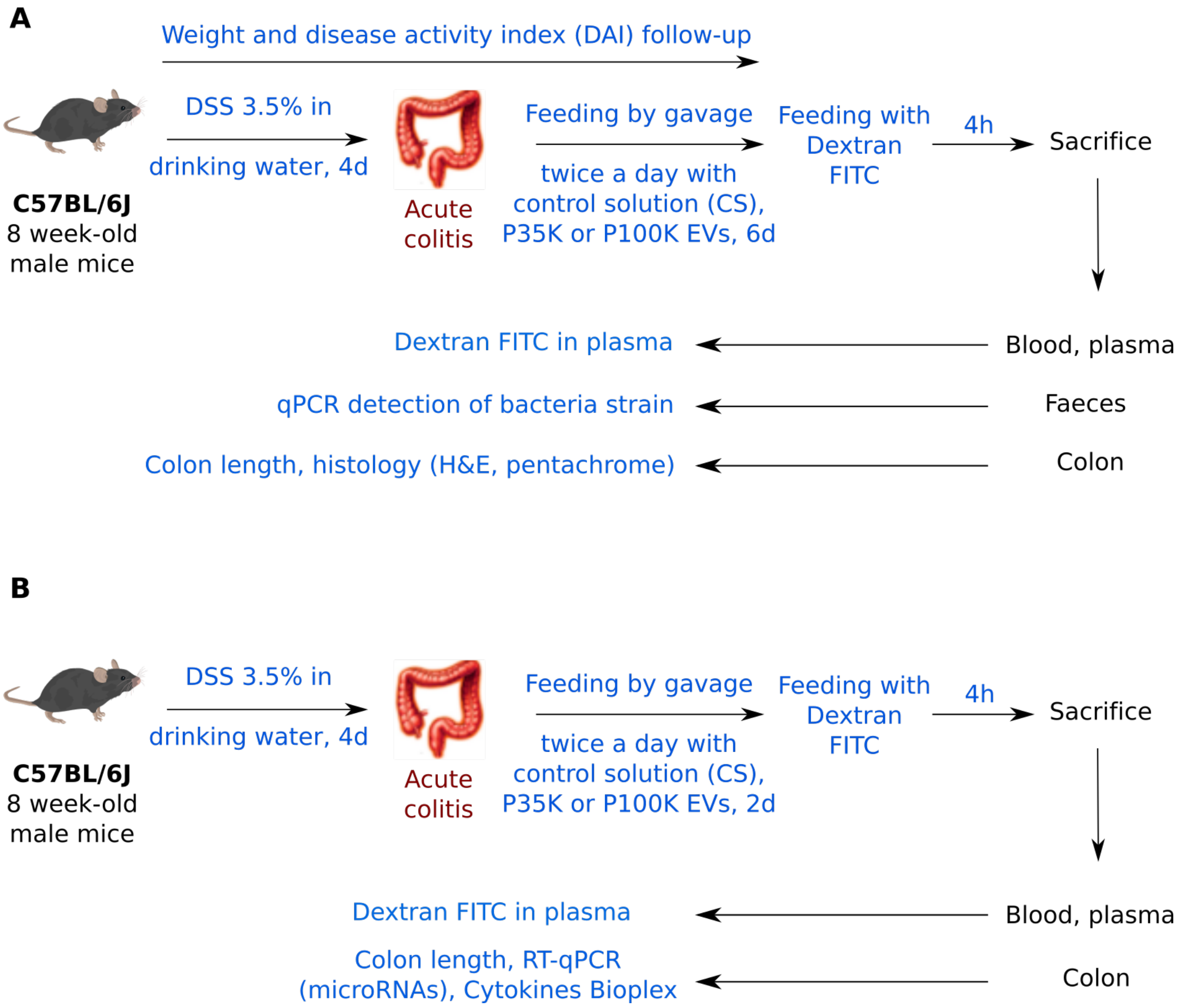

Supplementary Figure S1. Schematic representation of the experimental procedures. A. After 4 days of ulcerative colitis (UC) induction using DSS, mice were fed for 6 days with one of the three feeding solutions before the sacrifice, and the different parameters were measured. **B.** In the second round of the experiment, mice were fed only for 2 days with one of the three feeding solutions. Abbreviations. CS, control solution; DSS, Dextran Sodium Sulfate; d, day; EVs, Extracellular vesicles; FITC, fluorescein isothiocyanate; H&E, hematoxylin and eosin; P35K, Pellet 35 000 g ; P100K, Pellet 100,000g; UC, ulcerative colitis. The image of Acute colitis is from Ungaro et al.[1].

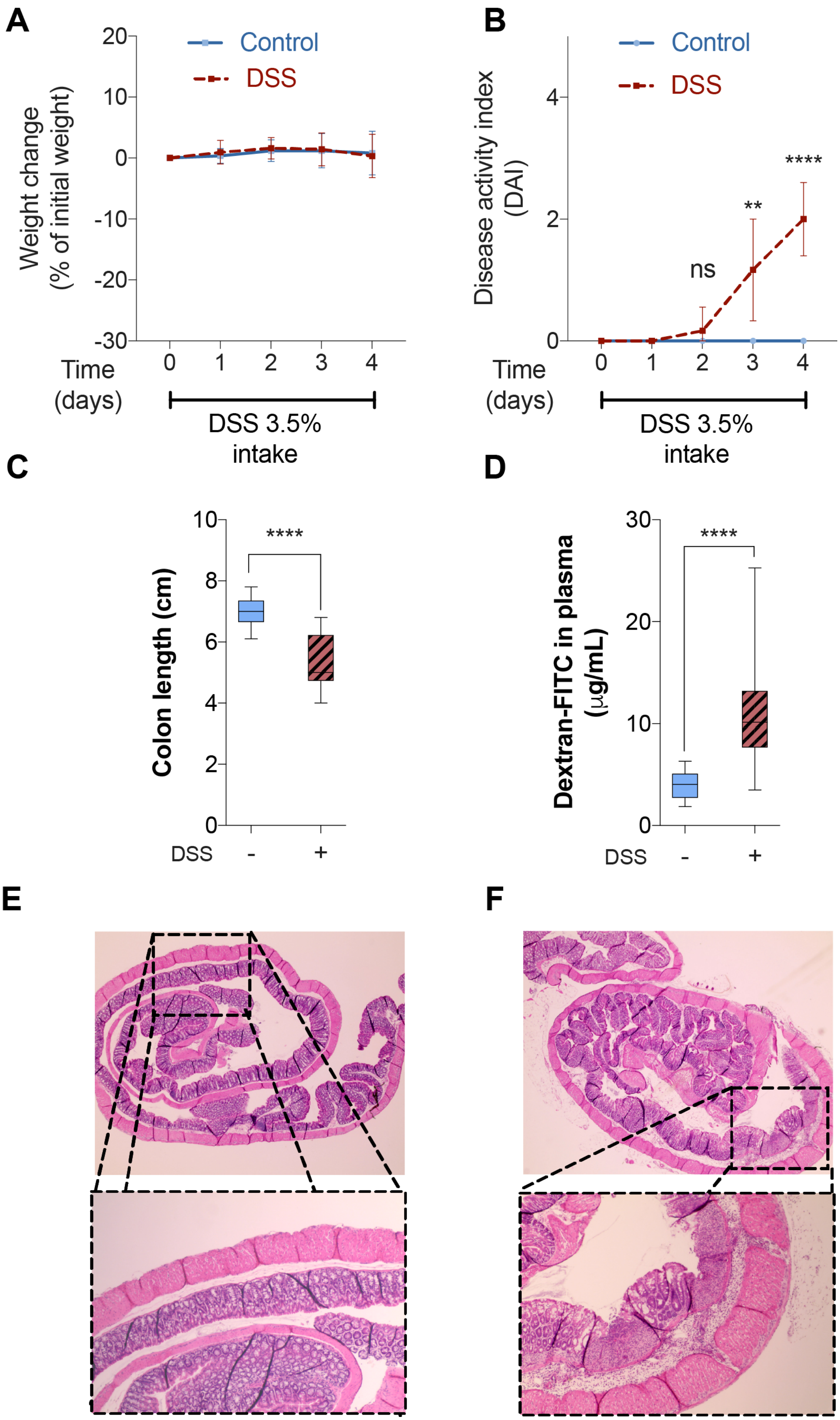

A
B
C
D
E
F
Weight change (% of initial weight)
Control
DSS
Time (days)
DSS 3.5% intake
Disease activity index (DAI)
ns
**
****
Colon length (cm)
Dextran-FITC in plasma (μg/mL)
DSS
-
+

Supplementary Figure S2. Setup of the mouse DSS-induced colitis model. Mice were exposed to DSS in their drinking water for 4 days. **A**. Mean weight change over time expressed as a percentage of the initial weight ± SD. **B**. DAI over time. Data are expressed as median ± 95% CI. **C**. Colon length in healthy and inflamed mice. **D**. Dextran-FITC quantification in plasma in healthy and colitic mice. E-F. Healthy (**E**) and inflamed (**F**) mice colon section colored with H&E (swiss roll). **Statistical comparison**. For weight, colon length and fluorescence in plasma, statistical significance was determined by one-way Anova with Bonferroni's post-hoc correction for multiple comparison. For DAI, statistical significance was determined by Kruskal-Wallis one-way analysis of variance with Dunn's Post-hoc correction for multiple comparison. For all tests, $p < 0.05$ was considered significant (n=12/group). **Significance display**. **** $p < 0.0001$; ns, not significant. Abbreviations. CI, confidence interval; DAI, disease activity index; DSS, dextran sodium sulfate; FITC, fluorescein isothiocyanate; SD, standard deviation.

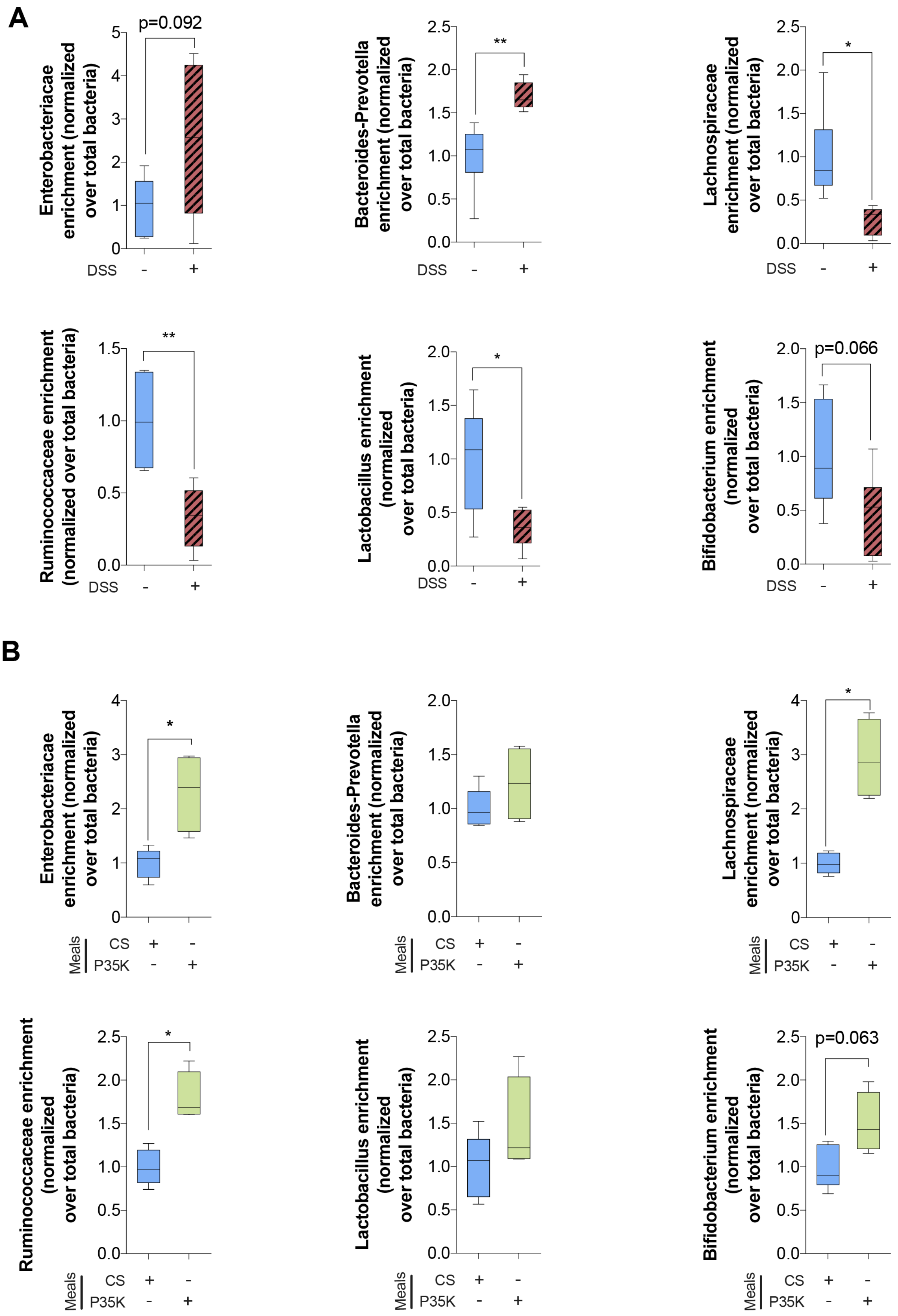
A
Enterobacteriacae enrichment (normalized over total bacteria)
p=0.092
DSS - +
Bacteroides-Prevotella enrichment (normalized over total bacteria)
**
DSS - +
Lachnospiraceae enrichment (normalized over total bacteria)
*
DSS - +
Ruminococcaceae enrichment (normalized over total bacteria)
**
DSS - +
Lactobacillus enrichment (normalized over total bacteria)
*
DSS - +
Bifidobacterium enrichment (normalized over total bacteria)
p=0.066
DSS - +
B
Enterobacteriacae enrichment (normalized over total bacteria)
*
Meals CS + -
P35K - +
Bacteroides-Prevotella enrichment (normalized over total bacteria)
Meals CS + -
P35K - +
Lachnospiraceae enrichment (normalized over total bacteria)
*
Meals CS + -
P35K - +
Ruminococcaceae enrichment (normalized over total bacteria)
*
Meals CS + -
P35K - +
Lactobacillus enrichment (normalized over total bacteria)
Meals CS + -
P35K - +
Bifidobacterium enrichment (normalized over total bacteria)
p=0.063
Meals CS + -
P35K - +

Supplementary Figure S3. Ingestion of DSS and P35K EVs influence colonic bacterial levels after 4 and 14 days, respectively. A. Mice were exposed to DSS in drinking water for 4 days before the sacrifice. Relative quantification of 6 colonic bacterial families by RT-qPCR. Data are expressed as fold change versus healthy mice. Bacteroidetes and Enterobacteriacae levels increased after disease induction, while the other strains were less abundant. **B.** Mice were fed twice a day with a preparation of P35K EVs (each meal corresponding to the EVs from 10 mL of commercial cow's milk). Relative quantification of 6 colonic bacterial families by RT-qPCR. Data are expressed as fold change versus CS-fed mice. Enterobacteriacae, Lachnospiracae and Ruminococaccae levels increased when mice were fed with P35K EVs. **Statistical comparison.** Statistical significance was determined by two-way Mann-Witney test with p value <0.05 considered significant (n=6 for panel **A**, n=5 for panel **B**). **Significance display.** * p <0.05; ** p <0.01. **Abbreviations.** CS, control solution; DSS, dextran sodium sulfate; P35K, pellet 35,000 g.

Supplementary Table S1. RT-qPCR primers used to quantitate bacterial strains. Adapted from Fernandez et al.[2].

Group	Primer	Sequence	T°C	Reference
**Total bacteria**	**Forward**	ACTCCTACGGGAGGCAGCAG	**55**	3
	**Reverse**	ATTACCGCGGCTGCTGG		
**Bacteroides—Prevotella group**	**Forward**	GAAGGTCCCCCACATTG	**55**	4
	**Reverse**	CGCKACTTGGCTGGTTCAG		
**Lactobacillus / Pediococcus / Leuconostoc spp.**	**Forward**	AGCAGTAGGGAATCTTCCA	**55**	5
	**Reverse**	CGCCACTGGTGTTCYTCCATATA		
**Enterobacteriaceae**	**Forward**	CATTGACGTTACCCGCAGAAGAAGC	**55**	6
	**Reverse**	CTCTACGAGACTCAAGCTTGC		
**Bifidobacterium spp.**	**Forward**	ATCTTCGGACCBGAYGAGAC	**55**	7
	**Reverse**	CGATVACGTGVACGAAGGAC		
**Ruminococcaceae (Clostridium cluster IV)**	**Forward**	TTAACACAATAAGTWATCCACCTGG	**55**	4
	**Reverse**	ACCTTCCTCCGTTTTGTCAAC		
**Lachnospiraceae (Clostridium cluster XIVa)**	**Forward**	AAATCACGGTACCTGACTAA	**55**	8
	**Reverse**	CTTTGAGTTCATTCTTGCGAA		

MISEV2018 CHECKLIST

Numbers refer to sections listed in the Table of contents from: C. Théry and K.W.Witwer, et al, "Minimal Information for Studies of Extracellular Vesicles 2018 (MISEV2018): a position statement of the International Society for Extracellular Vesicles and update of the MISEV2014 guidelines", J Extracell Vesicles 2018;7:1535750.

+++ Mandatory   ++ Mandatory if applicable   + Encouraged

1-Nomenclature

Mandatory

+++ Generic term extracellular vesicle (**EV**): **With demonstration** of **extracellular** (no intact cells) and **vesicular** nature per these characterization (Section 4) and function (Section 5) guidelines

In this study, we used the generic term EVs and previous characterization guidelines published in the Journal of Extracellular Vesicles demonstrating the vesicular nature of the particles whose function we investigated here.

Encouraged (choose one)

+ Generic term extracellular vesicle (**EV**) + **specification** (size, density, other)

We defined the compared EVs following their sedimentation at either 35,000 (P35K) or 100,000 g (P100K).

+ Specific term for subcellular origin: e.g., ectosome, microparticle, microvesicle (from plasma membrane), exosome (from endosomes), **with demonstration** of the subcellular origin

We found, in previous studies, that the commercial cow's milk EVs sedimenting at 100,000 g are very close to exosomes in nature, but we rather keep naming them 100K EVs for the sake of consistency.

+ Other specific term: **with definition of specific criteria**

N/A

2-Collection and pre-processing

Tissue Culture Conditioned medium (CCM, Section 2-a)

N/A

Biofluids or Tissues (Sections 2-b and -c)

++ Donor status if available (age, sex, food/water intake, collection time, disease, medication, other)

A pool of three milks with different expiration dates and from commercial origin (local grocery in Quebec City) from a pool a cows. Commercial cow's skim milk, pasteurized and filtered (Lactantia brand, Purefiltre).

+++ Volume of biofluid or volume/mass of tissue sample collected per donor

EVs isolated from 10 mL per mice per feeding (suspended in 100 uL of vehicle solution for feeding).

++ Total volume/mass used for EV isolation (if pooled from several donors)

See the Methods section.

+++ All known collection conditions, including additives, at time of collection

Milk was bought on the day of the experiment.

+++ Pre-treatment to separate major fluid-specific contaminants before EV isolation

Milk was mixed with 2% sodium citrate in a 1:1 ratio.

+++ Temperature and time of biofluid/tissue handling before and during pre-treatment

Kept at 4°C during the entire process.

++ For cultured tissue explants: volume, nature of medium and time of culture before collecting conditioned medium

N/A

++ For direct tissue EV extraction: treatment of tissue to release vesicles without disrupting cells

N/A

Storage and recovery (Section 2-d)

+++ Storage and recovery (e.g., thawing) of CCM, biofluid, or tissue before EV isolation (storage temperature, vessel, time; method of thawing or other sample preparation)

Milk was kept at 4°C, without freeze-thaw cycles.

+++ Storage and recovery of EVs after isolation (temperature, vessel, time, additive(s)…)

EVs were stored in vehicle solution (see Methods section), stored at 4°C overnight before feeding to mice.

3-EV separation and concentration

Experimental details of the method

++ Centrifugation: reference number of tube(s), rotor(s), adjusted k factor(s) of each centrifugation step (= time+ speed+ rotor, volume/density of centrifugation conditions), temperature, brake settings

The samples were subjected to successive differential ultracentrifugation steps at 35,000 g (35K) for 2 h, then 70,000 g for 1 h, and 100,000 g (100K) for 1 h at 4°C in a Sorvall WX TL-100 ultracentrifuge, equipped with a SureSpin 630 Rotor (Sorvall). Automated calculations of the K factors. Break A=9, D=9.

++ Density gradient: nature of matrix, method of generating gradient, reference (and size) of tubes, bottom-up (sample at bottom, high density) or top-bottom (sample on top, low density), centrifugation speed and time (with brake specified), method and volume of fraction recovery

N/A

++ Chromatography: matrix (nature, pore size,…), loaded sample volume, fraction volume, number

N/A

++ Precipitation: reference of polymer, ratio vol/vol or weight/vol polymer/fluid, time/temperature of incubation, time/speed/temperature of centrifugation

N/A

++ Filtration: reference of filter type (=nature of membrane, pore size…), time and speed of centrifugation, volume before/after (in case of concentration)

N/A

++ Antibody-based : reference of antibodies, mass Ab/amount of EVs, nature of Ab carrier (bead, surface) and amount of Ab/carrier surface

N/A

++ Other…: all necessary details to allow replication

Make sure to slowly resuspend the pellets to avoid forming EV aggregates.

++ Additional step(s) to concentrate, if any

N/A

++ Additional step(s) to wash matrix and/or sample, if any

EVs from 100 mL were rinsed in 100 mL of PBS (overnight suspension and spin again at the same ultracentrifugation speeds, i.e. 35,000 or 100,000 g).

Specify category of the chosen EV separation/concentration method (Table 1):

We used Intermediate recovery, intermediate specificity method = mixed EVs with limited non-EV components with milk citration limiting protein aggregation and sedimentation of non-EV components.

4-EV characterization

Quantification (Table 2a, Section 4-a)

+++ Volume of fluid, and/or cell number, and/or tissue mass used to isolate EVs

10 mL per mice per feeding

+++ Global quantification by at least 2 methods: protein amount, particle number, lipid amount, expressed per volume of initial fluid or number of producing cells/mass of tissue

One dose (200 µL) of each feeding solution corresponded to EVs isolated from 10 mL of commercial cow's milk (~430 mg/kg body weight). We choose to express this as mg per body weight as these EVs were fed and the control groups had to be fed with the same protein concentration. Moreover, we did not have access to a NTA, and the high-sensitivity flow cytometer we have cannot quantify EVs below 90 nm and would have underestimated the number of EVs. On the course of 6 months, our method yielded systematically very close protein concentration for each pellet.

+++ Ratio of the 2 quantification figures

N/A (see previous)

Global characterization (Section 4-b, Table 3)

Characterization was performed in two previous publications[9,10] following MISEV recommendations and using complementary approaches.

Single EV characterization (Section 4-c)

Characterization was performed in two previous publications[9,10] following MISEV recommendations and using complementary approaches.

5-Functional studies

+++ Dose-response assessment

We compared only one dose of EVs during a time-course experiment and compared two EV subsets in vivo, thereby limiting dose-response analysis.

+++ Negative control = nonconditioned medium, biofluid/tissue from control donors, as applicable

We used the supernatant of the last ultracentrifugation (100,000 g), which we further depleted from its EV content by a 100,000 g ultracentrifugation at 4°C during 18 h. The supernatant was then

diluted to match the protein content of milk EVs.

+++ Quantitative comparison of functional activity of total fluid, vs EV-depleted fluid, vs EVs (after high recovery/low specificity separation)

We compared EVs to depleted fluid. We did not compare with total fluid, because we used concentrated EVs, whose concentration is not comparable to the entire fluid (supraphysiological therapeutic concentrations).

+++ Quantitative comparison of functional activity of EVs vs other EPs/fractions after low recovery/high specificity separation

Such approaches do not yield enough EVs to study the effect of their activity during 7 days feeding twice a day in vivo, while keeping EVs fresh from the day. We will investigate these aspects in further studies in vitro.

+ Quantitative comparison of activity of EV subtypes (if subtype-specific function claimed)

We compared the effect of two EV subsets.

+ Extent of functional activity in the absence of contact between EV donor and EV recipient

N/A

6-Reporting

+ Submission of methodologic details to EV-TRACK (evtrack.org) with EV-TRACK number provided (strongly encouraged)

We will submit the details to EV-TRACK once the publication process is completed. We provide hereby most of the details in this supplementary file.

+++ Submission of data (proteomic, sequencing, other) to relevant public, curated databases or open-access repositories

All data are provided.

+ Data submission to EV-specific databases (e.g., EVpedia, Vesiclepedia, exRNA atlas)

N/A

++ Temper EV-specific claims when MISEV requirements cannot be entirely satisfied (Section 6-b)

We made sure to clarify the nature of the isolate we analyzed. Our analysis and characterization (7 different methods) strongly support the vesicular nature and relative purity of the EVs we analyzed[9,10].

SUPPLEMENTARY REFERENCES

1. Ungaro, R., Mehandru, S., Allen, P. B., Peyrin-Biroulet, L. & Colombel, J. F. Ulcerative colitis. *Lancet* **389**, 1756-1770, doi:10.1016/S0140-6736(16)32126-2 (2017).
2. Fernandez, B., Savard, P. & Fliss, I. Survival and Metabolic Activity of Pediocin Producer Pediococcus acidilactici UL5: Its Impact on Intestinal Microbiota and Listeria monocytogenes in a Model of the Human Terminal Ileum. *Microbial ecology*, doi:10.1007/s00248-015-0645-0 (2015).
3. Guo, X. *et al.* Development of a real-time PCR method for Firmicutes and Bacteroidetes in faeces and its application to quantify intestinal population of obese and lean pigs. *Letters in applied microbiology* **47**, 367-373, doi:10.1111/j.1472-765X.2008.02408.x (2008).
4. Ramirez-Farias, C. *et al.* Effect of inulin on the human gut microbiota: stimulation of Bifidobacterium adolescentis and Faecalibacterium prausnitzii. *The British journal of nutrition* **101**, 541-550, doi:10.1017/s0007114508019880 (2009).
5. Furet, J. P. *et al.* Comparative assessment of human and farm animal faecal microbiota using real-time quantitative PCR. *FEMS microbiology ecology* **68**, 351-362, doi:10.1111/j.1574-6941.2009.00671.x (2009).
6. Bartosch, S., Fite, A., Macfarlane, G. T. & McMurdo, M. E. T. Characterization of bacterial communities in feces from healthy elderly volunteers and hospitalized elderly patients by using real-time PCR and effects of antibiotic treatment on the fecal microbiota. *Applied and environmental microbiology* **70**, 3575-3581, doi:10.1128/AEM.70.6.3575-3581.2004 (2004).
7. Cleusix, V., Lacroix, C., Dasen, G., Leo, M. & Le Blay, G. Comparative study of a new quantitative real-time PCR targeting the xylulose-5-phosphate/fructose-6-phosphate phosphoketolase bifidobacterial gene (xfp) in faecal samples with two fluorescence in situ hybridization methods. *Journal of Applied Microbiology* **108**, 181-193, doi:10.1111/j.1365-2672.2009.04408.x (2010).
8. Matsuki, T. *et al.* Development of 16S rRNA-gene-targeted group-specific primers for the detection and identification of predominant bacteria in human feces. *Appl Environ Microbiol* **68**, 5445-5451 (2002).
9. Benmoussa, A. *et al.* A subset of extracellular vesicles carries the bulk of microRNAs in commercial dairy cow's milk. *Journal of extracellular vesicles* **6**, 1401897, doi:10.1080/20013078.2017.1401897 (2017).
10. Benmoussa, A., Gotti, C., Bourassa, S., Gilbert, C. & Provost, P. Identification of protein markers for extracellular vesicle (EV) subsets in cow's milk. *J Proteomics* **192**, 78-88, doi:10.1016/j.jprot.2018.08.010 (2019).
